# Electroconvulsive therapy use for refractory status epilepticus in an implantable vagus nerve stimulation patient: A case report

**DOI:** 10.3389/fpsyt.2023.1126956

**Published:** 2023-02-03

**Authors:** Lauren Katzell, Emily M. Beydler, Richard Holbert, Laura Rodriguez-Roman, Brent R. Carr

**Affiliations:** ^1^College of Medicine, University of Florida, Gainesville, FL, United States; ^2^Department of Psychiatry, University of Florida, Gainesville, FL, United States

**Keywords:** electroconvulsive therapy, status epilepticus, vagus nerve stimulation, refractory epilepsy, case reports

## Abstract

**Introduction:**

Status epilepticus (SE) has a mortality rate of 20 to 50%, with acute symptomatic SE having a higher risk compared to chronic SE. Electroconvulsive therapy (ECT) has been utilized for the treatment of refractory SE with a success rate estimate of 57.9%. There are no known reported cases of concomitant use of vagus nerve stimulation (VNS) and ECT for the treatment of super refractory SE (SRSE) available in the literature.

**Case description:**

We present a 44-year-old female with a history of developmental delay, epilepsy, an implantable VNS for 6 years, and traumatic brain injury with subsequent hygroma who presented with progressive aphasia, declining mental status, and daily generalized seizures lasting up to 20 min. Seizures had increased from her baseline of one seizure per day controlled with topiramate 200 mg three times daily and lamotrigine 400 mg twice daily. She was diagnosed with SRSE after being intubated and placed on eight anti-epileptic drugs (AEDs) that failed to abort SE. ECT was attempted to terminate SE. Due to a prior right craniotomy with subsequent right hygroma, eight treatments of ECT were performed over three sessions using a right anterior, left temporal (RALT) and subsequently a bitemporal electrode placement. The VNS remained active throughout treatment. Various ECT dosing parameters were attempted, varying pulse width and frequency. Although ECT induced mild transient encephalographic (EEG) changes following ECT stimulations, it was unable to terminate SE.

**Discussion:**

This case describes various treatment strategies, constraints, and device limitations when using ECT for the treatment of SE. With wide variability in efficacy rates of ECT in the treatment of SE in the literature, successful and unsuccessful cases offer information on optimizing ECT total charge dose and parameters that yielded success. This case demonstrates an instance of ECT inefficacy in the treatment of SRSE. Here, we discuss the rationale behind the various ECT settings that were selected, and constraints arising from the antiepileptic burden, VNS, and intrinsic limitations of the ECT device itself.

## Introduction

Status epilepticus (SE) has a mortality rate purported to be 20 to 50%, with acute symptomatic SE having a higher risk compared to chronic SE (1). Electroconvulsive therapy (ECT) has been utilized for the treatment of SE when traditional therapies fail, however, success rates of ECT in cases of super refractory SE (SRSE) have not been well-documented. In 2012, the success rate of ECT in the treatment of SE was reportedly 80%, however, an analysis in 2016 demonstrated the rate as 57.9% (2, 3). While the mechanism behind the efficacy of ECT in SE remains unclear, proposed mechanisms include the release of inhibitory transmitters, such as GABA; prolongation of the refractory period; elevation of the seizure threshold, which has been demonstrated in patients receiving ECT for treatment of mood disorders; and induction of endogenous seizure termination mechanisms (1, 3). Vagus nerve stimulation (VNS) has been approved for use in drug-resistant epilepsy in the United States since 1997 and has been shown to reduce SE occurrence and its recurrence (4). VNS interrupted refractory SE in 74% of patients, with a median duration of 8 days post-implantation for cessation (4). Most reported cases of concomitant use of VNS with ECT detail circumstances in which the VNS was turned off prior to ECT treatment. There are no known reported cases of concomitant use of VNS and ECT in treatment of SRSE available in the literature. Here we present a case of the use of ECT to treat SRSE in a patient with an active VNS.

## Case description

**FIGURE 1:**
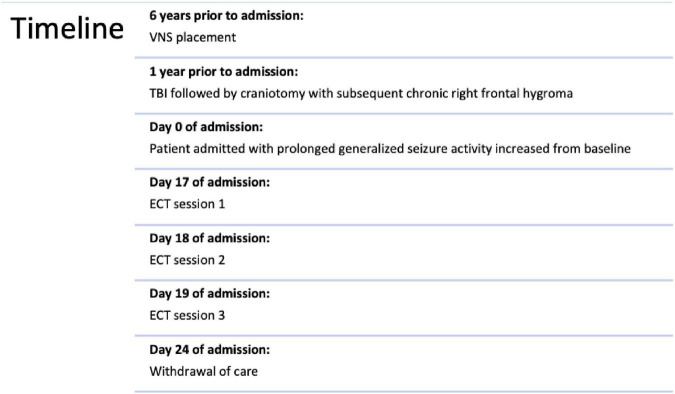
Timeline of clinical events.

Here we present a 44-year-old female with a history of developmental delay, localization-related epilepsy diagnosed at age 16, VNS placement at age 38, and status post-head injury with intracranial bleed that required craniotomy 1 year prior who presents with daily prolonged periods of generalized seizures lasting up to 20 min that had increased from her baseline of one seizure per day previously controlled by her home regimen of topiramate 200 mg TID and lamotrigine 400 mg BID (Figure 1). Upon admission, she was experiencing progressive aphasia and declining mental status. Lumbar puncture revealed HHV-6 encephalitis for which she was started on foscarnet. She was subsequently intubated and due to medication refractory SE, started on an increasingly large antiepileptic regimen including lamotrigine, levetiracetam, topiramate, Perampanel, clobazam, pregabalin, cannabidiol, phenobarbital, propofol drip, and ketamine drip.

She was determined to not be a candidate for a ketogenic diet. Ultimately, the multidisciplinary team decided to attempt ECT to terminate the SRSE. The VNS remained on throughout the three-session ECT treatment course, with parameters set at 1.75 mA, 250 μs pulse width, 30 s on, and 1.1 min off time. The risk of concurrent treatment in patients with a VNS implant arises from the use of strong electromagnetic fields such as seen with diathermy (i.e., short-wave diathermy, microwave diathermy, or therapeutic ultrasound diathermy), where such treatment anywhere in the body could potentially lead to injury *via* heating or damage the implanted VNS stimulator, even when the VNS is turned off (5). This includes electromagnetic fields seen with transcranial magnetic stimulation. Even so, the VNS Therapy System is safe for use in 1.5 and 3 T MRI scanners (6). However, ECT creates an electrical field within the brain, and heating *via* diathermy would not be expected. The VNS uses an implanted pulse generator in the chest connected to an electrode in the neck outside the ECT electrical field. Similarly, numerous case reports have shown the safe use of ECT in those with deep brain stimulation implants that are within the electrical field produced with ECT (7). Given that the VNS impulses that reach the tractus solitarius arise from the implanted pulse generator in the periphery, the creation of thermal injury was not expected (5, 6). Although turning the VNS device current to zero during ECT is the most customary practice (8), we were constrained to proceed with VNS on given intensive care unit (ICU) concerns of worsening the seizure burden in a SRSE patient should the device be turned off.

**FIGURE 2:**
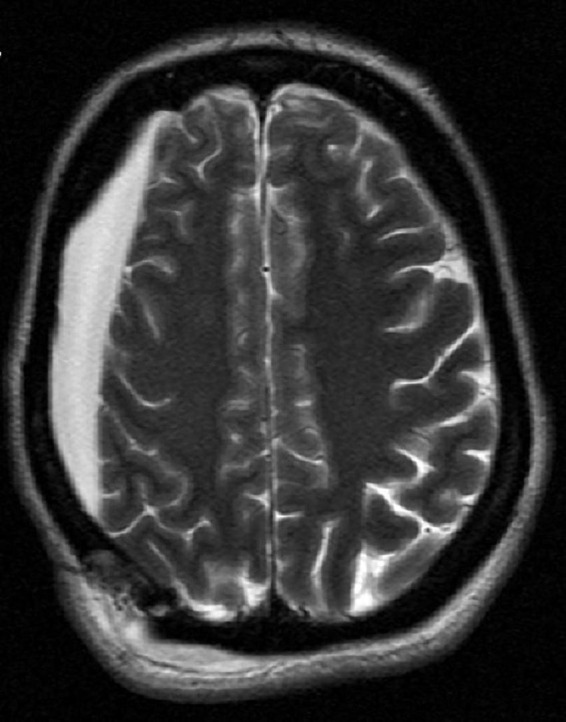
Magnetic resonance imaging showing patient’s right hygroma prior to initiation of electroconvulsive therapy (ECT).

Due to the patient’s prior right craniotomy and subsequent right hygroma (Figure 2), initial ECT sessions were performed with a right anterior, left temporal (RALT) lead placement using a MECTA spECTrum 5000Q ECT Device (9). To maximize cortical recruitment and depolarization to break the SRSE, with or without induction of a seizure, ECT session 1 (total charge dose: 2.0 ms, 3 s, 120 Hz, 800 mA) was performed on day 17 with propofol and ketamine drips paused 30 min before treatment. SE continued so an attempt to induce a seizure with more contemporary parameter settings as seen in psychiatric ECT was utilized. Here the patient was restimulated twice at 60-s intervals using more efficient settings for seizure induction with an ultra-brief pulse width and longer stimulus train to induce a seizure (0.37 ms, 6 s, 120 Hz, 800 mA). ECT session 1 was unable to induce seizures nor halt her baseline seizure activity, and seizure burden continued to increase from 11 to 20% burden over the next 24 h. ECT session 2 was performed with the intensivist team finally agreeing on day 18 to pause ketamine and propofol infusions 2 h prior to the session, having previously been reluctant to do so for fear of worsening SE. Session 2 consisted of four stimulations each separated by 60 s, with the first two using 1 ms, 6 s, 60 Hz, 800 mA and the latter two stimulations using 2 ms, 3 s, 60 Hz, 800 mA. Shorter pulse widths were not used as these settings would have prevented 100% of the total device energy that was to be used. ECT session 2 induced transient epileptiform activity with burst suppression but was then followed by an almost immediate return to baseline SE. Seizure burden continued to increase to 30% despite a combination of treatments. ECT session 3 was performed on day 19, now with ketamine and propofol infusions paused 3 h before the session. It consisted of one stimulus at 0.5 ms, 3 s, 60 Hz, 800 mA with a 3-min hiatus followed by 1 ms, 3 s, 60 Hz, 800 mA. The final stimulus attempted to maximize interelectrode distance with bitemporal lead configurations (Table 1). ECT session 3 again induced mild epileptiform activity with subsequent return to baseline without meaningful change in seizure burden post-ECT stimulus. The VNS remained on during ECT as the primary team was reluctant to turn the device off; VNS interrogation following ECT treatments showed its proper functioning. In discussion with the epileptologist and neurologic critical care team, as the seizure burden had continued to steadily worsen over the course of the patient’s illness, and with only mild transient encephalographic (EEG) changes from ECT treatment, the decision was made to discontinue ECT treatment. The patient’s family opted for comfort care measures, and the patient died on day 24.

**TABLE 1:** Electroconvulsive therapy (ECT) series parameters.

	Pulse width (ms)	Duration (s)	Frequency (Hz)	Amplitude (mA)	Configuration
Session 1	2.0	3	120	800	Right anterior, left temporal (RALT)
	0.37	6	120	800	RALT
	0.37	6	120	800	RALT
Session 2	1	6	60	800	RALT
	1	6	60	800	RALT
	2	3	60	800	RALT
	2	3	60	800	RALT
Session 3	0.5	6	60	800	RALT
	1	3	60	800	Bitemporal

## Discussion

When ECT is used in mood disorders, techniques are employed to lower the seizure threshold such as inducing hypocarbia with hyperventilation or the use of proconvulsant administration prior to an ECT session. However, such strategies become problematic when trying to balance the use of ECT in a patient that is in SE, where the lowering of seizure threshold could worsen the underlying condition. There is wide variability in efficacy rates of ECT in the treatment of SE, and, problematically, unsuccessful cases often go unreported. Moreover, eliciting a seizure in the context of SE is often difficult, primarily due to the typically large anticonvulsant pharmacological load used in treating these patients, such as coma induction *via* barbiturate, and for our patient the anti-epileptogenic implantable VNS, as well as her other antiepileptics, all of which hinder seizure induction through ECT. Should the goal have been to stimulate a large volume of brain to abolish a seizure, analogous to electrical cardioversion of electrical dysrhythmias? Or should the aim be to induce a seizure itself that endogenously and spontaneously resolves, thus terminating the underlying status; or is it through raising the seizure threshold itself by repeated ECT treatments that underlies its efficacy? Without clear answers to these questions, we attempted all strategies. Information on the positioning of the stimulating electrodes, total ECT charge, and parameter breakdown yielding termination of SE are often sparsely documented. The rise in seizure threshold that is seen in a classic Index Series of ECT with multiple sessions can be leveraged for the treatment of SE. However, it is unclear if the rates of rise in seizure threshold is similar for SE patients, but repeated ECT sessions have led to termination of SE (10, 11).

In the classic use of ECT for mood disorders, seizure quality parameters are often observed to deem seizure quality. Such data might include information such as post-ictal suppression index, time to peak coherence, and energy after an ECT treatment. Here we are unable to provide equivalent information on induced epileptiform activity as seen with the typical ECT treatments for two reasons. First, we were unable to induce seizures long enough that would have been interpretable by the ECT device. Second, our intensive care unit patient already had continuous long-term EEG monitoring. We employed MECTA’s ECT stimulus electrodes for delivery of the stimulus but did not attach the MECTA ECT device’s two, two lead EEG montage, rather observing the stimulus results with the 21 scalp electrodes using the International 10–20 System recommended by the International Federation of Clinical Neurophysiology (IFCN). The digital EEG operated continuously at the patient’s bedside. Standard digital video EEG techniques were used throughout, including computerized spike and seizure detection with a technologist review *in situ*. The digital analysis methodology was interpreted by the ICU’s epileptologist who used quantitative EEG analysis where long term trending was performed including compressed density spectral array and spectral measures of rhythmicity, symmetry, power, amplitude, and alpha/beta ratio.

For this case, initial settings were selected that were deemed the most likely to create a large volume depolarization event, endeavoring to break status, even should induction of a seizure not occur. Although the decision to use the longer pulse width prior to an ultra-brief pulse in this trial was seemingly arbitrary; however, it was assumed it would be difficult to elicit a seizure given the intravenous antiepileptic burden and the active VNS. It was hoped that one stimulation with a large volume depolarization event, maximizing cortical recruitment, might fortuitously terminate the SE irrespective of whether a quality ECT seizure had been induced (as would desirable when treating refractory mood disorders). This would obviate the need for further ECT, including the multiple sessions that might be required to try to raise the seizure threshold to achieve termination of SE. However, it was also unclear if this strategy of using a longer 2 ms pulse width initially might transiently interfere with the threshold, making it more difficult to induce a seizure on immediately subsequent stimulation if needed should SE not terminate (as was the case). In consideration of this, on subsequent days the order of longer pulse width preceding the shorter was reversed (see Table 1).

The initial pulse width selected, 2 ms, theoretically would recruit axons that are both larger and smaller in diameter (12, 13). It should be noted that the 2 ms pulse width has been abandoned in contemporary ECT treatments for the initial treatment of mood disorders as this parameter selection is remarkably inefficient at seizure induction, given that it exceeds the chronaxie for neuronal depolarization throughout most of the cortex (14). Of note, this is contrary to some case reports that have shown that such settings with high cortical volume involvement were the only way seizure induction had been possible, including when more efficient parameter settings of brief pulse widths had previously failed (13, 14). For our case, the long pulse width failed to terminate the SE and did not induce a generalized seizure.

After the first stimulation, a 1-min repolarization hiatus was taken, and we transitioned to briefer pulse widths and longer stimulus trains. These theoretically were more efficient parameters for seizure induction. Here, the rationale was to induce a generalized seizure, thereby potentially terminating SE as the induced seizure subsided. An equally valid approach for improving the efficiency of seizure induction may have been to use a lower frequency on the device, allowing greater repolarization between paired pulses. However, the lower frequency would require longer stimulation trains, interfering with the ability to achieve maximum device output for our ECT device. Such low frequency and brief pulse widths are the standard approach in contemporary ECT and allow for more efficient seizure induction at a lower total device charge by avoiding stimulus crowding (15).

Initial electrode placement was RALT. This placement was used to avoid the region with underlying cortical pathology, i.e., the chronic hygroma. RALT allowed for reasonable interelectrode distance. This is opposed to an idealized placement of the stimulating electrodes immediately over a solitary seizure focus, which is impractical given the extreme shunting of electricity through the scalp that would arise through reducing the interelectrode distance. Such shunting and current spreading *via* the scalp, and the skull’s high resistance, during electrical stimulation is a known problem that can interfere with ECT. Novel approaches for seizure induction such as Magnetic Seizure Therapy attempt to resolve this issue (16). When multiple attempts at this placement failed, we proceeded with bitemporal ECT despite its positioning the electrical field bilaterally across the cortex, and over the hygroma. This placement was selected to maximize interelectrode distance in an attempt to minimize interelectrode shunting through the scalp. Unfortunately, bitemporal ECT also failed to terminate status in this patient.

It should be noted that, during ECT for depression, the implantable VNS device is typically turned off to avoid interference with ECT from its antiepileptic effects. Moreover, to diminish interference with seizure threshold during ECT, anesthesia induction agents have been optimized to have the least impact. Moreover, there is conflicting information regarding the impact of anti-epileptic drugs (AEDs) on the efficacy of ECT in mood disorders. Such reports typically cite a reduced seizure duration in patients on AEDs which might suggest reduced seizure efficacy. However, this effect seems less pronounced after a first induced seizure is obtained, and newer guidelines recommend against discontinuation of AEDs prior to ECT initiation (17).

There were a few limitations that hindered our ability to elicit seizure. One was the understandable reluctance of the primary team to lower agents that might allow for easier seizure induction due to fear of worsening SE. Moreover, our patient was on multiple AEDs rather than a single drug, with doses designed to suppress seizure activity. This undoubtedly contributed to our difficulty in inducing a seizure. It is also unclear to what extent such AEDs would affect an ECT induced seizure’s ability to increase seizure threshold in SE. However, reports show efficacy of ECT in patients on who are on multiple AEDs (18, 19). After the initial ECT session failed to produce a seizure, antiepileptic infusions were held prior to subsequent ECT sessions. In these sessions, ECT-induced brief epileptiform. Reducing propofol dosing further or turning off the implantable VNS would potentially have given ECT a greater chance of success than the more conservative strategy that presumably hampered any potential gains from ECT. Regarding the concurrent use of ECT with her active VNS, VNS interrogation revealed proper functioning throughout and after ECT.

Another serious limitation was the device constraint for the allowed total charge that may be delivered. For psychiatric ECT, it is known that a successful response is correlated with the magnitude of the seizure EEG discharge and subsequent inhibitory processes. This response magnitude is correlated with some minimum degree to which the seizure threshold is superseded. It is plausible that merely inducing a seizure may be inadequate for driving the seizure threshold upward for SE. This need to supersede threshold is constrained for the ECT devices used in the United States and Canada where the maximal charge is limited at either 504 or 576 mC, depending on which device is used (20, 21). The same devices in much of the world have double this limit and would afford a marked theoretical advantage (22). Available stimulus output was noted to be insufficient for 5% of patients, which could represent the most severe cases, such as those with SRSE, such that there is a call for an increase in maximum stimulus output for ECT devices (20). In support of this need for larger device output is a university research group (who introduced the use of ECT in SE with good success) using research devices with three times the contemporary United States limit (21).

Unfortunately, ECT was ineffective in aborting this patient’s SE. Plausible causes contributing to this failure are cited above. However, ECT has been shown to be successful in terminating SE. And, with well-documented reporting of both positive and negative outcomes, and the ECT techniques that are used, better outcomes can be achieved. This will aid in the establishment of treatment guidelines and suggestions for approaching ECT in SE. Such suggestions would include AED reduction strategies, determining optimal parameter selection and electrode placements, and exploration of mitigation strategies for the United States/Canadian devices with their less efficient total charge dosing.

## Data availability statement

The original contributions presented in this study are included in this article/supplementary material, further inquiries can be directed to the corresponding author.

## Ethics statement

Ethical review and approval was not required for the study on human participants in accordance with the local legislation and institutional requirements. The patients/participants provided their written informed consent to participate in this study. Written informed consent was obtained from the individual(s) for the publication of any potentially identifiable images or data included in this article.

## Author contributions

LK and BC wrote first and final drafts of the manuscript. EB and RH assisted in draft revisions. LR-R performed manuscript review. All authors approved the submitted version.
